# Further Exploring the Public Health Implications of the Network Scale-Up Method: Cross-Sectional Survey Study

**DOI:** 10.2196/48289

**Published:** 2024-08-23

**Authors:** Liwei Jing, Hongmei Yu, Qing Lu

**Affiliations:** 1 Department of Health Statistics, School of Public Health, Shanxi Medical University, 56 South XinJian Road, Taiyuan, 030001, China, 86 03514135049; 2 Department of Epidemiology and Biostatistics, Michigan State University, East Lansing, MI, United States

**Keywords:** network scale-up method, public health implications, people who inject drugs, popularity ratio, information transmission rate, PWID

## Abstract

**Background:**

The decline in the number of new HIV infections among adults has slowed down, gradually becoming the biggest obstacle to achieving the 2030 target of ending the HIV/AIDS epidemic. Thus, a political declaration to ensure that 90% of people at high risk of HIV infection can access comprehensive prevention services was proposed by the United Nations General Assembly. Therefore, obtaining an accurate estimated size of high-risk populations is required as a prior condition to plan and implement HIV prevention services. The network scale-up method (NSUM) was recommended by the United Nations Programme on HIV/AIDS and the World Health Organization to estimate the sizes of populations at high risk of HIV infection; however, we found that the NSUM also revealed underlying population characteristics of female sex workers in addition to being used to estimate the population size. Such information on underlying population characteristics is very useful in improving the planning and implementation of HIV prevention services. This is especially relevant for people who inject drugs, where in addition to stigma and discrimination, criminalization further hinders access to HIV prevention services.

**Objective:**

We aimed to conduct a further exploration of the public health implications of the NSUM by using it to estimate the population size, popularity ratio, and information transmission rate among people who inject drugs.

**Methods:**

A stratified 2-stage cluster survey of the general population and a respondent-driven sampling survey of people who inject drugs were conducted in the urban district of Taiyuan, China, in 2021.

**Results:**

The estimated size of the population of people who inject drugs in Taiyuan was 1241.9 (95% CI 1009.2‐1474.9), corresponding to 4.4×10^−2^% (95% CI 3.6×10^−2^% to 5.2×10^−2^%) of the adult population aged 15‐64 years. The estimated popularity ratio of people who inject drugs was 53.6% (95% CI 47.2%‐60.1%), and the estimated information transmission rate was 87.9% (95% CI 86.5%‐89.3%).

**Conclusions:**

In addition to being used to estimate the size of the population of people who inject drugs, the NSUM revealed that they have smaller-sized personal social networks while concealing their drug use, and these underlying population characteristics are extremely useful for planning appropriate service delivery approaches with the fewest barriers for people who inject drugs to access HIV prevention services. Therefore, more cost-effectiveness brings new public health implications for the NSUM, which makes it even more promising for its application.

## Introduction

In the past 15 years, the successful scale-up of antiretroviral therapy has effectively controlled the number of AIDS-related deaths. Furthermore, continued efforts to eliminate mother-to-child transmission of HIV have led to a significant decline in the number of new HIV infections among children each year. Therefore, global progress against HIV and AIDS has made it possible to end the epidemic by 2030 [1]. However, the decline in the number of new HIV infections among adults has slowed down, gradually becoming the biggest obstacle to achieving the 2030 target [1]. Between 2010 and 2022, new HIV infections declined by 35% in the 15‐ to 49-year-old population globally, but only by 11% among people at high risk of HIV infection [2,3]. Discrimination, stigma, and criminalization hinder the access and availability of services for people at high risk of HIV infection [2]. Thus, a political declaration to ensure that 90% of people at high risk of HIV infection can access comprehensive prevention services was proposed by the United Nations General Assembly in 2016 [1]. Therefore, obtaining an accurate estimated size of high-risk populations is required as a prior condition to plan and implement HIV prevention services [4,5]. As the latest size estimation method of high-risk populations recommended by the Joint United Nations Programme on HIV/AIDS (UNAIDS) and the World Health Organization (WHO) in 2010, the network scale-up method (NSUM) is a more cost-effective method compared to all of the previous size estimation methods because it produces size estimates for multiple high-risk populations using a single survey [4,6]. For example, the sizes of the populations of female sex workers, people who inject drugs, and men who have sex with men could be estimated through a single NSUM survey.

The NSUM was first proposed by Bernard et al [7] in 1991 and uses social network information of the general population to estimate the sizes of populations at high risk of HIV infection. In 2011, Salganik et al [8,9] used the popularity ratio and information transmission rate to adjust for the barrier effects and transmission error that existed in the NSUM. In 2018, we found that these 2 coefficients revealed underlying population characteristics of female sex workers in addition to being used to adjust the estimated population size [10]. Populations at high risk of HIV infection are considered hidden or hard-to-reach populations because of their stigmatized nature; therefore, such information on underlying population characteristics is very useful in improving the planning and implementation of HIV prevention services [10]. This is especially relevant for people who inject drugs, since an overwhelming majority of countries (143 countries) criminalized the use or possession of small amounts of drugs [11]. Thus, in addition to stigma and discrimination, criminalization further hinders access to HIV prevention services for people who inject drugs. As a result, global coverage of HIV prevention services is extremely low, with less than 1% of people who inject drugs living in settings with sufficient, combined, and high-coverage services [11]. This resulted in 38.1% of people who inject drugs not knowing their HIV status [12]; thus, the risk of acquiring HIV infection for people who inject drugs was 35 times higher than for people who do not inject drugs [13]. Globally, HIV infection rates have declined across all age groups, while HIV infection rates in people who inject drugs have been rising [14].

The prevalence of injecting drug use varied greatly both between and within countries [15]. Globally, almost half of the people who inject drugs live in China, Russia, and the United States [14]. In China, people who inject drugs are concentrated in 7 provinces (Yunnan, Xinjiang, Guangxi, Guangdong, Guizhou, Sichuan, and Hunan). Taiyuan, the capital of Shanxi Province, is not from a province with a high prevalence of injecting drug use, but it represents the situation in most cities in China [16]. Thus, this study aimed to conduct a further exploration of the public health implications of the NSUM by using it to estimate the population size, popularity ratio, and information transmission rate of people who inject drugs in the urban district of Taiyuan, China, in 2021.

## Methods

### The NSUM

Female sex workers, people who inject drugs, and men who have sex with men are considered high-risk populations, are members of the general population, and live in the social network of the general population. Therefore, the average proportion of high-risk populations in the personal social network of the general population also reflects the proportion of high-risk populations in the general population in the region. Thus, the basic formula of the NSUM is *e_1_ / t = m_1_ / c*, where *e_1_* is the estimated size of high-risk populations in the region, *t* is the latest annual average population in the region, *m_1_* is the reported average number of high-risk population members in the personal social network of each respondent from the general population, and *c* is the reported average number of members in the personal social network of each respondent from the general population [6,17,18]. Membership in a personal social network was defined using the following statement: “They know you and you know them by name or by sight, they live in this region, and you have had some contact with them in the past 12 months” [18]. The average size of the personal social networks of the general population (*c*) can be calculated by another formula: *e_0_ / t = m_0_ / c*, where *e_0_* is the sum of a list of specific populations, for which the actual size of the population is known by researchers (McCormick et al [19] verified that recall bias can be minimized through the approach of using given last names, with each accounting for 0.1%‐0.2% of the urban population in a region, as a known population to estimate the average size of the personal social network); *t* is the latest annual average population in the region; and *m_0_* is the average size of the reported specific populations in the personal social network of each respondent from the general population [20,21].

However, there are 2 situations that could affect NSUM estimates. First, the average size of the personal social networks of high-risk populations may be smaller because of their hidden nature; therefore, their probability of becoming members of the personal social networks of the general population may be reduced. These barrier effects could cause the NSUM to underestimate the size of high-risk populations. Second, members in the personal social network of high-risk populations may be unaware that these people engage in high-risk behaviors [4,8,9,22]. This transmission error could also cause underestimation. In 2011, Salganik et al [8,9] proposed the popularity ratio and information transmission rate to adjust for the barrier effects and transmission error, respectively. The popularity ratio is the ratio of the average size of the personal social network of the high-risk population to the average size of the personal social network of the general population. The information transmission rate is the ratio of the average number of members in the personal social network of the high-risk population who are aware that these people engage in high-risk behaviors to the average size of the personal social network of the high-risk population. Finally, the NSUM estimate (*e_1_*) is divided by the popularity ratio and information transmission rate to adjust for the barrier effects and transmission error, respectively.

### Field Survey

The NSUM was applied to estimate the population size, popularity ratio, and information transmission rate of people who inject drugs in the urban district of Taiyuan, China. Taiyuan is the capital city of Shanxi Province in northern China and has a population of 4.5 million. The field survey consisted of 2 parts, including a stratified 2-stage cluster survey of the general population to obtain *e_1_* and a respondent-driven sampling (RDS) survey of people who inject drugs to obtain the popularity ratio and information transmission rate. RDS is a standard method recommended by the WHO to obtain representative samples of people at high risk of HIV infection [23-25]. All surveys were conducted between May and October 2021.

### Stratified 2-Stage Cluster Survey of the General Population

A stratified 2-stage cluster survey of the general population was conducted in respondents’ workplaces. According to the sample size required for stratified 2-stage cluster sampling, the sample size of the general population survey was 1600 respondents [26]. In the first stage, 36 primary units (institutions, organizations, and companies) from all 20 industries (agriculture, manufacturing, electricity, construction, transportation, information transmission, retail, accommodations and catering, finance, real estate, business services, and others) were selected, with the probability of selection being proportional to the industry size from the sampling frame. In the second stage, 83 second-stage units (departments) were selected from the primary units chosen in the first stage. Finally, 1679 third-stage units (individual respondents) from the departments chosen in the second stage were interviewed in their workplaces. In addition, 68 unemployed respondents were interviewed at their former workplaces or communities. All respondents were required to be at least 18 years old.

In the survey, respondents were asked the following: (1) the number of members in their personal social network whose last names are on a given list of last names, with each last name accounting for 0.1% to 0.2% of the population of Taiyuan (48 last names were used in our survey), and (2) the number of people who inject drugs in their personal social network. People who inject drugs are defined as people aged 15 years or older who had injected drugs (ie, illicit, nonprescribed, or illegal substances) at least once in the past 12 months [27]. Because question 2 is sensitive, an unrelated-question randomized response technique was used to eliminate response bias [28]. Before the interview, respondents were randomly divided into 2 groups. The respondents of the first group randomly received question 2 (the number of people who inject drugs in their personal social network) or an unrelated question (average hours on the web per week), with the ratio of the 2 questions being 8:2. The ratio of the 2 questions for the second group was 2:8. Because only each respondent knows which question was answered, their privacy was protected, and question 2 was more likely to be answered truthfully. After the interview, the mean and variance of the respondent’s answer to question 2 were calculated by the following formula [28]:


$$\mu =\frac{\left(1-P_{2}\right)\times \mu_{1}-\left(1-P_{1}\right)\times \mu_{2}}{P_{1}-P_{2}}$$

where *μ*_1_ is the mean of the responses from the first group, *μ*_2_ is the mean of the responses from the second group, *P_1_* is the proportion of sensitive questions in the first group, and *P_2_* is the proportion of unrelated questions in the second group.

### RDS Survey of People Who Inject Drugs

The RDS survey of people who inject drugs began with 2 seeds. According to sentinel surveillance data of drug users in Taiyuan (unpublished data, 2024), heroin and methamphetamine account for more than 90% of all drugs injected; thus, 1 user of heroin and 1 user of methamphetamine were used as the 2 seeds. As the survey was conducted during the COVID-19 pandemic, people who inject drugs were interviewed at COVID-19 nucleic acid amplification testing sites. First, the screeners checked whether the respondents met all of the following criteria: (1) had injected in the past 12 months, (2) living in the urban district of Taiyuan, and (3) aged 18 years or older. Second, the respondents were asked the following: (1) the number of members in their personal social network whose last names are on a given list of 48 last names, with each last name accounting for 0.1% to 0.2% of the population of Taiyuan, and (2) the number of members with specific last names in their personal social network who are aware of their drug use. Third, each respondent received 3 coupons to recruit peers. After 8 waves, a total of 302 people who inject drugs were interviewed to estimate the popularity ratio and information transmission rate. Due to RDS, the bootstrap method (10,000 resamples) was applied to calculate the 95% CIs for the popularity ratio and information transmission rate [29-31].

### Ethical Considerations

The study was approved by the Medical Ethics Committee of Shanxi Medical University (reference 2018001). The study was conducted anonymously to protect the privacy of respondents. We received a waiver of the informed consent requirement for all respondents because no personally identifiable data were collected in the survey. All respondents were compensated for the time they spent participating in the survey (approximately US $7 each). In addition, unemployed respondents and respondents who inject drugs received transportation subsidies (approximately US $7 each).

## Results

### Results of the Stratified 2-Stage Cluster Survey in the General Population

**Figure 1.**
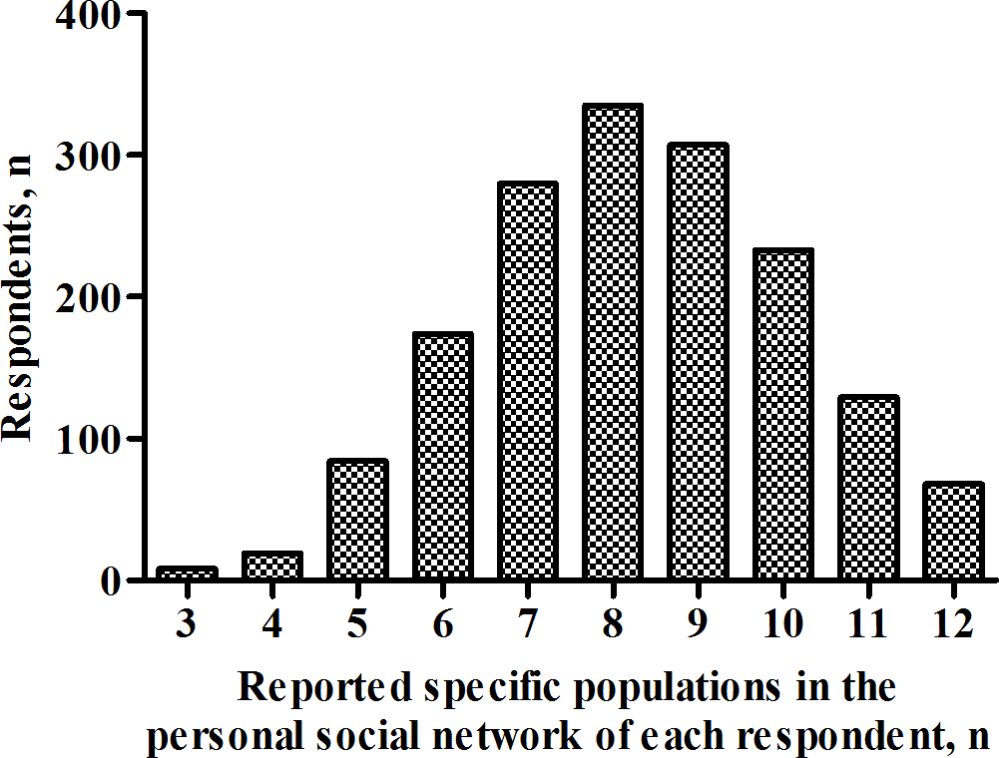
Size of specific populations in the personal social network of each respondent from the general population in Taiyuan, China, in 2021.

In the survey of the general population, despite the use of a randomized response technique, respondents who were still embarrassed during the survey were encouraged to submit unanswered answer sheets to ensure that they do not respond falsely. Overall, 6.3% (110/1747) of the respondents submitted unanswered answer sheets. Therefore, 93.7% (1637/1747) of respondents were successfully interviewed. The average size of the reported specific populations in the personal social network of each respondent (*m_0_*) was 8.3 (95% CI 6.2‐10.3; Figure 1). The sum of the 48 specific populations of given last names (*e_0_*=302,251) and the annual average population in Taiyuan in 2021 (*t*=4,531,429) were obtained before the survey; therefore, the estimated average size of the personal social network of the general population (*c*) in Taiyuan was 123.9 ([8.3×4,531,429]/302,251; 95% CI 93.0‐154.4). Furthermore, the reported average number of people who inject drugs in the personal social network of each respondent (*m_1_*) was 1.6×10^−2^ (95% CI 1.3×10^−2^ to 1.9×10^−2^); therefore, the initial NSUM estimate of the population size of people who inject drugs (*e_1_*) in Taiyuan was 585.1 ([1.6×10^−2^/123.9]×4,531,429; 95% CI 475.5‐694.9).

### Results of the RDS Survey of People Who Inject Drugs

**Figure 2.**
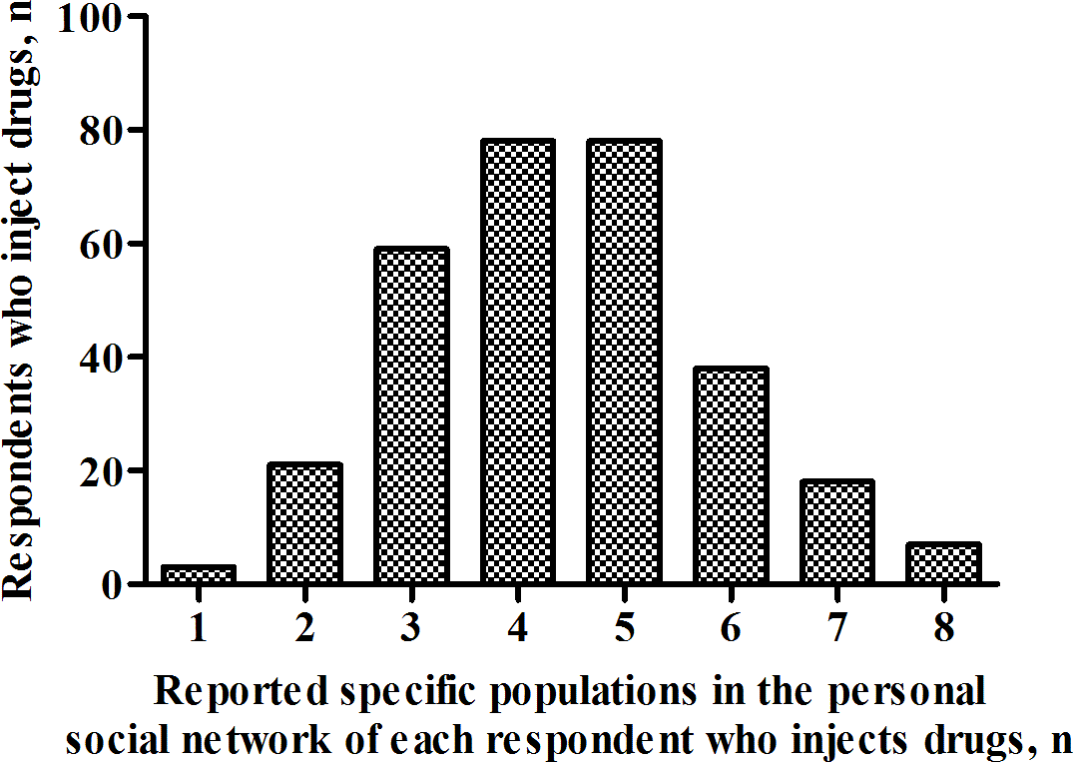
Size of specific populations in the personal social network of each respondent who injects drugs in Taiyuan, China, in 2021.

**Figure 3.**
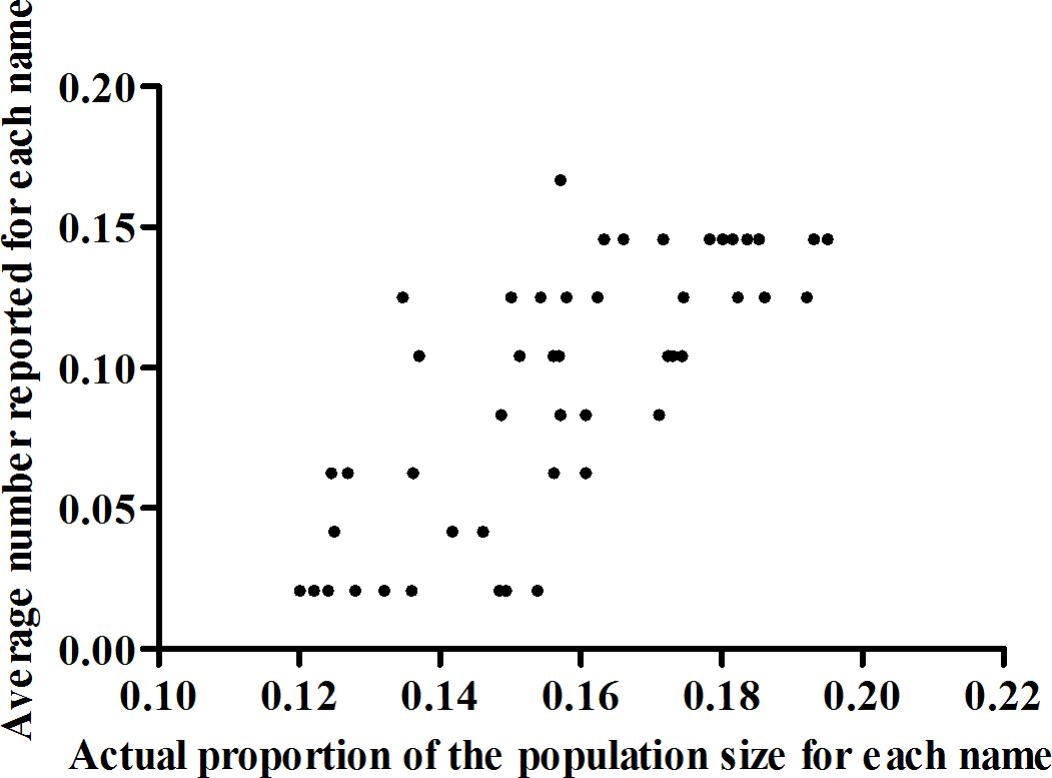
Reported proportion by respondents who inject drugs compared to the actual proportion of the population size for each name in Taiyuan, China, in 2021.

**Figure 4.**
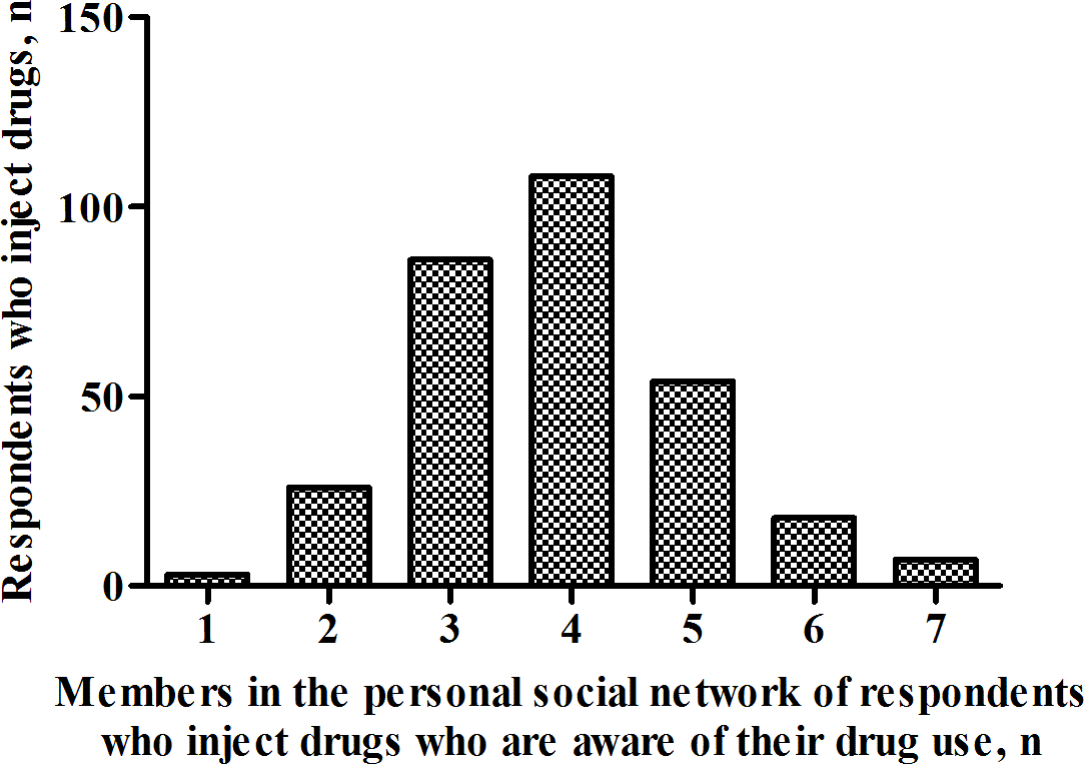
Number of members in the personal social network of respondents who inject drugs who are aware of their drug use in Taiyuan, China, in 2021.

The average reported size of the 48 specific populations of given last names in the personal social network of each person who inject drugs was 4.4 (95% CI 4.3‐4.6 [adjusted estimate]; Figure 2); this number strongly correlated with the actual proportion of the population size for each of the 48 last names (*r*=0.7, *P*<.001; Figure 3). Therefore, the estimated average size of the personal social network of people who inject drugs in Taiyuan was 66.3 ([4.4×4,531,429]/302,251; 95% CI 63.5‐69.2), and the estimated popularity ratio of people who inject drugs in Taiyuan was 53.6% (66.3/123.9; 95% CI 47.2%‐60.1%). In addition, the average reported number of members with the 48 specific last names in the personal social network of respondents who inject drugs who are aware of their drug use was 3.9 (95% CI 3.7‐4.0 [adjusted estimate]; Figure 4); therefore, the estimated average number of members in the personal social network of people who inject drugs who are aware of their drug use was 58.5 ([3.9×4,531,429]/302,251; 95% CI 55.5‐60.0), and the estimated information transmission rate of people who inject drugs in Taiyuan was 87.9% (58.5/66.3; 95% CI 86.5%‐89.3%).

### Results of the Adjusted NSUM Estimate

Finally, the adjusted NSUM estimate of the population size of people who inject drugs in Taiyuan was 1241.9 (585.1/[53.6%×87.9%]; 95% CI 1009.2‐1474.9), corresponding to 4.4×10^−2^% (1241.9/2,820,541; 95% CI 3.6×10^−2^% to 5.2×10^−2^%) of the adult population aged 15‐64 years. In China, people who inject drugs are concentrated in 7 provinces (Yunnan, Xinjiang, Guangxi, Guangdong, Guizhou, Sichuan, and Hunan) [16]. Therefore, the NSUM estimate of the population proportion of people who inject drugs (0.044%) in our study in Shanxi Province was below the national-level estimate (2.5×10^−1^%, 95% CI 1.9×10^−1^% to 3.1×10^−1^%) obtained from a recent worldwide systematic review of the proportion of adults aged 15‐64 years who inject drugs in 2008 [15].

## Discussion

### Principal Findings

The NSUM was recommended by UNAIDS and WHO to estimate the sizes of the populations at high risk of HIV infection. However, we found that the NSUM also revealed underlying population characteristics of people who inject drugs. In this study, the average size of the personal social network of people who inject drugs was only 53.6% of the average size of the personal social network of the general population. Barrier effects thus reduced the opportunity for people who inject drugs to be members of the personal social network of the general population by 46.4% because of the hidden nature of this population, with its smaller average personal social network size. Additionally, 87.9% of the members of the personal social network of people who inject drugs were aware of their drug use. Transmission error thus increased the possibility of people who inject drugs concealing their drug use from personal social networks by 12.1% because of the stigmatized nature of this population. Therefore, the popularity ratio and the information transmission rate together revealed characteristics of the population of people who inject drugs: they have smaller-sized personal social networks while concealing their drug use. A similar situation has occurred in NSUM research, where the popularity ratio and information transmission rate were estimated. For example, in 2011, estimates of the popularity ratio and information transmission rate of people who inject drugs in Brazil were 0.69 and 0.77, respectively; in 2013, estimates for people who inject drugs in Iran were 0.69 and 0.54, respectively, and estimates for female sex workers were 0.77 and 0.45, respectively; and in 2018, estimates for female sex workers in China were 0.41 and 0.80, respectively [9,10,32].

However, such population characteristics present barriers to accessing HIV prevention services for people who inject drugs. This may partly explain the fact that few countries have achieved adequate coverage of HIV prevention services [1]. As a result, progress in preventing the spread of HIV remains far too slow. It was estimated that the total number of new infections worldwide in 2019 was more than 3 times higher than the 500,000 milestone set for 2020 [33]. Therefore, UNAIDS recommended that the situation should be carefully evaluated before providing HIV prevention services to improve the program’s effectiveness and sustainability [11]. In our study, the characteristics of people who inject drugs revealed by the NSUM indicated that peer-led services might be more effective than professional-led services in improving the coverage of HIV prevention services. For example, the comprehensive package for HIV prevention could be delivered by peer outreach workers (ie, individual members of the population of people who inject drugs who are trained to deliver or support access to harm reduction services) rather than health facilities. Because of a common experience, peer outreach workers are more likely to be trusted by people who inject drugs to give acceptable and appropriate HIV prevention services. Especially for HIV testing services, people who inject drugs could conduct self-testing counseled by peer outreach workers. This strategy is both discreet and convenient while improving disruptions in HIV prevention services for people who inject drugs caused by the COVID-19 pandemic because of health facilities repurposed to handle the influx of patients with COVID-19.

### Limitations

In general, household surveys are recommended for surveys of the general population. We piloted a household survey in this study. Unfortunately, the response rate was less than 10% due to the COVID-19 lockdowns. Therefore, we conducted a survey of the general population in respondents’ workplaces. According to the 2020 National Economic and Social Development Statistical Communiqué released on March 18, 2021, the unemployment rate in Taiyuan in 2020 was 3.2% [34], and thus, 68 unemployed respondents were interviewed at their former workplaces or communities. Although we included respondents who were unemployed proportionally, there was still some possibility of selection bias. In addition, due to the sensitive questions included in the survey, general information about the respondents’ characteristics (eg, age, gender, etc) was not collected in the survey. Therefore, we were unable to describe the general characteristics of the respondents in this study.

### Conclusions

As the latest method for size estimation of high-risk populations recommended by UNAIDS and WHO, in addition to producing size estimates for the population of people who inject drugs, the NSUM also revealed the social network characteristics of this population. These underlying population characteristics are extremely useful for planning appropriate service delivery approaches with the fewest barriers to access HIV prevention services for people who inject drugs. Therefore, the NSUM could help to improve the coverage of HIV prevention services, effectively preventing new HIV infections. More cost-effectiveness brings new public health implications for the NSUM, which makes it even more promising for its application.

## Abbreviations

NSUM: network scale-up method
RDS: respondent-driven sampling
UNAIDS: Joint United Nations Programme on HIV/AIDS
WHO: World Health Organization
